# The use of the ipsilateral dorsalis pedis artery approach for transarterial embolization in symptomatic chronic plantar fasciitis: a case report

**DOI:** 10.1093/jscr/rjaf1018

**Published:** 2025-12-23

**Authors:** Mohmmed Margni, Babatunde Almaroof, Fayad Mohamed, Ahmed Rafei

**Affiliations:** Vascular Institute of Michigan, 1325 S Linden RdFlint, MI 48532, United States; Vascular Institute of Michigan, 1325 S Linden RdFlint, MI 48532, United States; Vascular Institute of Michigan, 1325 S Linden RdFlint, MI 48532, United States; Vascular Institute of Michigan, 1325 S Linden RdFlint, MI 48532, United States

**Keywords:** plantar fasciitis, transcatheter arterial embolization, dorsalis pedis access, chronic heel pain

## Abstract

Plantar fasciitis is the most common cause of heel pain, and while most cases respond to conservative management, some remain refractory to treatment. Chronic cases are associated with abnormal neovascularization and increased fascial vascularity. Transcatheter embolization (TAE) has recently emerged as a minimally invasive alternative for musculoskeletal pain, though prior plantar fasciitis cases have primarily used femoral or posterior tibial access. We report the first case of chronic, treatment-resistant plantar fasciitis successfully managed with TAE via the ipsilateral Dorsalis Pedis artery. A 68-year-old male (BMI 40.46 kg/m^2^) underwent targeted embolization of hypervascular medial plantar branches using Imipenem–Cilastatin (2 mL). The procedure was completed in under 15 minutes, with minimal blood loss and no complications. The ipsilateral Dorsalis Pedis approach enables more precise targeting and reduced radiation exposure compared with posterior tibial access, while offering fewer complications and faster recovery compared with femoral access—representing a novel and effective option.

## Introduction

Plantar fasciitis is the most common cause of heel pain among patients presenting to outpatient clinics [1]. It accounts for approximately one million medical visits each year and represents about 10% of running-related injuries and 11%–15% of all foot disorders requiring professional medical attention [2]. The condition is more prevalent in women than in men, particularly among individuals aged 45–64 years compared with those aged 18–44 years, and in people with a BMI greater than 25 kg/m^2^ [3].

Pathophysiologically, plantar fasciitis is primarily an overuse injury resulting from recurrent strain that leads to microtears in the plantar fascia. However, trauma or multifactorial mechanical and systemic factors may also contribute to its development [1]. Common predisposing factors include pes planus, pes cavus, limited ankle dorsiflexion, prolonged standing or jumping, and excessive pronation or supination of the foot [4]. Over 90% of plantar fasciitis cases improve within 3–6 months of conservative management, but a small subset with persistent symptoms beyond 6–12 months may eventually require surgical intervention [5]. The chronic pain in these refractory cases is primarily attributed to abnormal neovascularization and increased plantar fascial vascularity [6].

Transcatheter arterial embolization (TAE) is a minimally invasive procedure that selectively occludes the abnormal neovessels supplying pathologic tissues. It has shown promising results in managing various musculoskeletal disorders, including osteoarthritis, tendinopathy, and enthesopathy—and more recently, in patients with plantar fasciitis [6]. Reported cases to date have primarily utilized femoral or posterior tibial artery access [6, 7], evidence from genicular artery embolization (GAE) procedures suggests that the pedal arterial approach provides better arterial targeting, reduced radiation exposure, fewer complications, and faster recovery [8]. Here, we present the first reported case of chronic plantar fasciitis successfully treated with TAE via Ipsilateral Dorsalis Pedis artery approach.

## Case presentation

A 68-year-old male (BMI 40.46 kg/m^2^) presented to our center for follow-up three weeks after undergoing GAE via the pedal approach for symptomatic right knee osteoarthritis. At follow-up, he reported complete resolution of knee pain (WOMAC Score = 2, reduced from a pre-procedure score of 47) and had discontinued all analgesics. However, following increased physical activity, he developed a severe flare-up of chronic left plantar fasciitis, characterized by lifestyle-limiting heel pain unresponsive to rest, orthotics, and NSAIDs.

His past medical history included hypertension, renal insufficiency, and kidney cancer treated with partial nephrectomy in 2019. Surgical history included tonsillectomy and hernia repair. The patient denied alcohol use, had quit smoking, and had no known drug allergies.

On examination, he was alert and in no acute distress. Vital signs were stable (T: 98°F, HR: 99 bpm, BP: 155/105 mmHg, SpO₂: 95%). Cardiopulmonary and abdominal examinations were unremarkable. Localized tenderness was noted at the medial calcaneal insertion of the left plantar fascia without swelling, ulceration, or skin changes. Neurological examination was normal.

**Figure 1:**
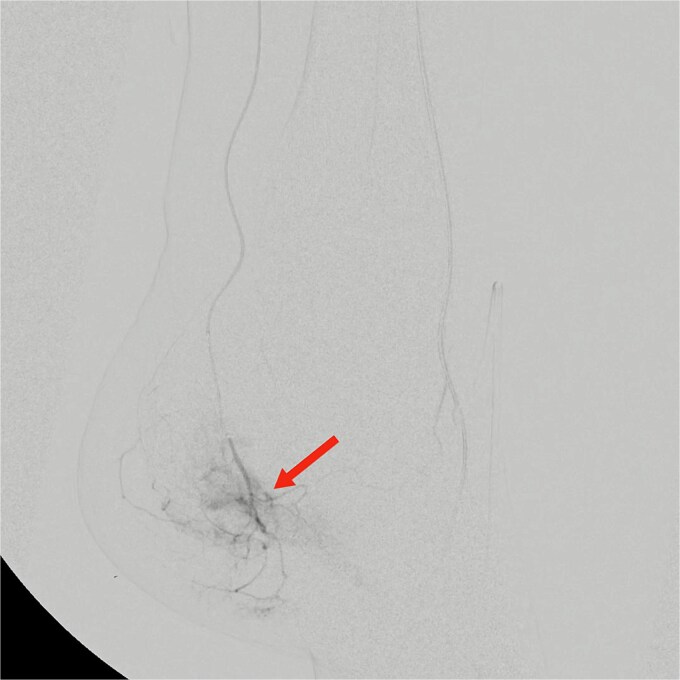
Pre-embolization digital subtraction angiography showing prominent hypervascular blush in branches of the medial plantar artery arising from the distal posterior tibial artery (arrow).

Given the refractory symptoms, TAE was planned. Under ultrasound guidance, the left dorsalis pedis artery was accessed retrogradely using a 5 Fr Cordis Rainsheath sheath (20 cm). After heparin administration, diagnostic angiography revealed patent anterior, posterior tibial, peroneal, and popliteal arteries. The tibioperoneal trunk was selected from the anterior tibial artery using a mini Rim catheter, followed by distal posterior tibial artery selection. Foot angiography demonstrated a prominent hypervascular blush in a branch of the medial plantar artery (Fig. 1).

**Figure 2:**
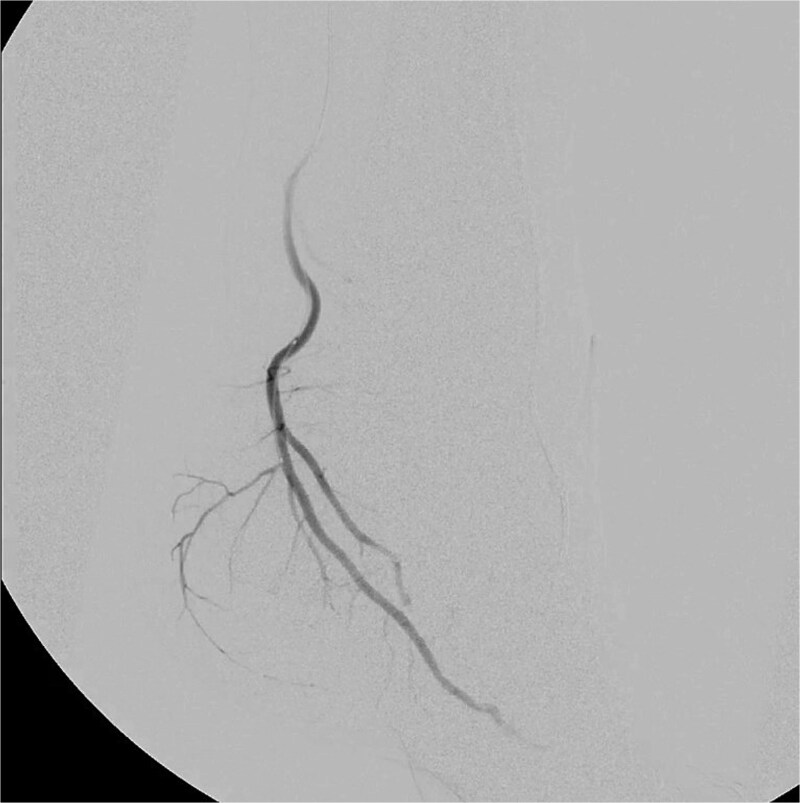
Selective catheterization and targeted embolization of the hypervascular branches of the left medial plantar artery using imipenem–Cilastatin (2 mL) as the embolic agent, demonstrating successful occlusion of abnormal vascularity on post-embolization angiography.

Selective catheterization of the target branch was performed using a 0.014-inch guidewire and microcatheter, followed by embolization with Imipenem–Cilastatin (2 mL) (Fig. 2). The vascular blush resolved completely, confirming procedural success. Hemostasis was achieved using a Safeguard compression device, with blood loss <30 mL and procedure time < 15 minutes. The total contrast used was 7 mL (Omnipaque, 200–299 mg I/mL).

Medications administered included lidocaine 1% (2 mL SC), heparin 500 U IV, nitroglycerin 25 μg IA, midazolam 2 mg IV, and fentanyl 100 μg IV. The patient remained stable throughout and experienced no complications.

He was discharged one hour post-procedure and reported immediate, complete pain relief. At two-week follow-up, he remained pain-free with full weight-bearing and normal ambulation. A three-month follow-up was scheduled for long-term evaluation.

## Discussion

TAE has emerged as a minimally invasive alternative to surgery in various chronic musculoskeletal conditions. In plantar fasciitis, its therapeutic concept relies on reducing abnormal vascularity within the inflamed fascia, thereby decreasing local inflammation and pain. By inducing controlled ischemia in hypervascular tissue, TAE limits the release of pro-inflammatory mediators and interrupts nociceptive signaling, offering durable symptom relief in patients unresponsive to conservative therapy [9].

Among available embolic agents, Imipenem–Cilastatin provides a favorable safety profile compared with traditional materials such as gelatin sponge. When suspended in contrast media, it achieves temporary and targeted occlusion, minimizing the risk of ischemic complications [9].

Pedal arterial access has been increasingly utilized in endovascular procedures, including GAE, demonstrating excellent technical success and superior arterial selectivity [8, 10]. This approach is particularly advantageous in obese patients, those with difficult groin anatomy, or individuals unable to lie completely flat—situations where femoral access may be challenging or risky [11].

By contrast, femoral access is associated with significantly higher complication rates, including hematoma (2%–12%), major bleeding (1.9%–14%), and pseudoaneurysm formation (0.5%–6.3%), with overall access-site complication rates reported up to 17% [12, 13]. These complications can delay recovery and increase morbidity, especially in patients with limited mobility or comorbidities.

Another described technique for plantar fasciitis involves direct puncture of the posterior tibial artery using a 24G indwelling needle [7]. While technically feasible, it places the operator’s hands directly in the fluoroscopic field, leading to radiation exposure without protective shielding and offering less precise vascular control.

In contrast, using the dorsalis pedis artery as the access site provides several procedural advantages. It allows selective catheterization of the distal posterior tibial and medial plantar artery branches for targeted embolization, ensuring precise treatment of the hypervascular area while avoiding non-selective embolization. The increased working distance also enables the placement of radiation shielding, thereby reducing operator exposure. Additionally, hemostasis can be achieved easily with devices like the Safeguard, resulting in faster recovery, early ambulation, and improved patient satisfaction.

## References

[1] Buchanan BK, Sina RE, Kushner D. Plantar Fasciitis. [Updated 2024 Jan 7]. In: StatPearls. Treasure Island (FL): StatPearls Publishing, 2025. 28613727

[2] Lapidus PW, Guidotti FP. Painful heel: report of 323 patients with 364 painful heels. Clin Orthop Relat Res 1965;39:178–86. 10.1097/00003086-196500390-00016 14289759

[3] Nahin RL . Prevalence and pharmaceutical treatment of plantar fasciitis in United States adults. J Pain 2018;19:885–96. 10.1016/j.jpain.2018.03.003 29597082 PMC6066406

[4] Mørk M, Soberg HL, Hoksrud AF, et al. The struggle to stay physically active-a qualitative study exploring experiences of individuals with persistent plantar fasciopathy. J Foot Ankle Res 2023;16:20. 10.1186/s13047-023-00620-4 37061709 PMC10105408

[5] Latt LD, Jaffe DE, Tang Y, et al. Evaluation and treatment of chronic plantar fasciitis. Foot Ankle Orthop 2020;5:2473011419896763. 10.1177/2473011419896763 35097359 PMC8564931

[6] Gandhi R, Banker M. Early outcomes of transcatheter arterial embolization using imipenem/cilastatin for plantar fasciitis refractory to conservative therapy. Br J Radiol 2024;97:544–8. 10.1093/bjr/tqae012 38281074 PMC11027232

[7] Shibuya M, Sugihara E, Miyazaki K, et al. Intra-arterial infusion of temporary embolic material in a patient with plantar fasciitis: a case report. Cardiovasc Intervent Radiol 2021;44:1823–6. 10.1007/s00270-021-02908-z 34231004

[8] Bagla S, Piechowiak R, Sajan A, et al. Multicenter randomized sham controlled study of genicular artery embolization for knee pain secondary to osteoarthritis. J Vasc Interv Radiol 2022;33:2–10.e2. 10.1016/j.jvir.2021.09.019 34610422

[9] Kishore S, Sheira D, Malin ML, et al. Transarterial embolization for the treatment of chronic musculoskeletal pain: a systematic review of indications, safety, and efficacy. ACR Open Rheumatol 2022;4:209–17. 10.1002/acr2.11383 34842365 PMC8916547

[10] Brocco E . The clinical utility of below-the-ankle angioplasty using “transmetatarsal artery access” in complex cases of CLI. Catheter Cardiovasc Interv 2013;83:123–9. 10.1002/CCD.24992 23696069

[11] Sajan A, Lerner J, Kasimcan MO, et al. Feasibility and technique of retrograde pedal access for genicular artery embolization. J Vasc Interv Radiol 2023;34:2030–3. 10.1016/j.jvir.2023.07.012 37495097

[12] Piper WD, Malenka DJ, Ryan TJ Jr, et al. Northern New England cardiovascular disease study group. Predicting vascular complications in percutaneous coronary interventions. Am Heart J 2003;145:1022–9. 10.1016/S0002-8703(03)00079-6 12796758

[13] Tsetis D . Endovascular treatment of complications of femoral arterial access. Cardiovasc Intervent Radiol 2010;33:457–68. 10.1007/s00270-010-9820-3 20162284

